# Understanding How Negative Emotions Affect Hazard Assessment Abilities in Construction: Insights from Wearable EEG and the Moderating Role of Psychological Capital

**DOI:** 10.3390/brainsci15020190

**Published:** 2025-02-13

**Authors:** Dan Chong, Siyu Liao, Mingjie Xu, Yuting Chen, Anni Yu

**Affiliations:** 1 School of Management, Shanghai University, Shanghai 200444, China; chongdan@shu.edu.cn (D.C.); lsy5189@shu.edu.cn (S.L.); mjxu@shu.edu.cn (M.X.); 15705732971@163.com (A.Y.); 2 Engineering Technology and Construction Management, The University of North Carolina at Charlotte, Charlotte, NC 28223, USA

**Keywords:** behavioral experiment, EEG emotion classification, negative emotion, psychological capital, unsafe behavior

## Abstract

**Background**: The construction industry faces significant safety hazards, frequent accidents, and inadequate management. Studies identify unsafe worker behaviors as the primary cause of construction accidents. However, most research overlooks the psychological state, particularly emotions, of construction workers. **Methods**: This study designed a behavioral experiment integrating social cognitive neuroscience, collecting real-time EEG data to classify and recognize fear, anger, and neutral emotions. Variance analysis explored differences in safety hazard identification and risk assessment under these emotional states. A total of 22 male participants were involved, with data collection lasting three days. The role of psychological capital in mediating the effects of emotions on unsafe behaviors was also examined. **Results**: Emotional classification using EEG signals achieved 79% accuracy by combining frequency domain and nonlinear feature extraction. Fear significantly enhanced safety hazard identification accuracy compared to neutral and anger emotions (F = 0.027, *p* = 0.03). Risk assessment values under fear and anger were higher than under neutral emotion (F = 0.121, *p* = 0.023). Psychological capital interacted significantly with emotions in hazard identification accuracy (F = 0.68, *p* = 0.034), response time (F = 2.562, *p* = 0.003), and risk assessment response time (F = 1.415, *p* = 0.026). Safety hazard identification correlated with the number of safety trainings (*p* = 0.002) and safety knowledge lectures attended (*p* = 0.025). Risk assessment was significantly associated with smoking (*p* = 0.023), alcohol consumption (*p* = 0.004), sleep duration (*p* = 0.017), and safety training (*p* = 0.024). **Conclusions**: The findings provide insights into how emotions affect safety hazard identification and risk assessment, offering a foundation for improving emotional regulation, reducing accidents, and enhancing safety management in construction.

## 1. Introduction

The construction industry is a vital component of the global economy, with the number of construction workers accounting for 4.1–9.1% of the total global workforce [1]. However, increasing safety accidents and fatalities highlight the need for urgent measures in the construction industry, such as strengthening safety management systems [2]. A complex environment, unique work tasks, temporary nature, and non-repeatability characterize the construction industry. As a result, the number of safety accidents is much higher than that of many other industries [3,4,5,6]. The main cause of safety accidents in the construction industry is the unsafe behavior of construction workers [7,8]. Emotions play a critical role in influencing workers’ safety behaviors, as they significantly influence cognitive processes such as hazard identification and risk assessment [9]. This study aims to explore how these emotions, measured through EEG signals, impact safety performance in the construction industry.

Existing research has shown that construction workers’ inability to quickly and accurately identify safety hazards often leads to unsafe behaviors [9]. There are various factors that influence the unsafe behaviors of construction workers, such as emotions, safety cognition, safety attitude, and safety motivation. Among these, the emotional state of individuals is closely related to their safety cognition, safety attitude, and motivation [10]. When performing tasks, workers cannot always keep rational thinking and are usually in a state of “bounded rationality”. Workers often make irrational decisions due to emotional drive [11], which is closely related to occupational health, safety, and job performance [12]. Changes in the organizational context and management factors can interfere with workers’ emotions, leading to unsafe behaviors [13].

Emotion is one of the processes of human mental activities and is an intuitive response to exposure to the surrounding environment. The existing research on emotion classifications has three classical theories, namely Plutchik’s theory, Ekman’s theory, and James Lange’s theory [10,14,15]. These theories categorize emotions into three types: positive emotions, negative emotions, and neutral emotions. Based on these classifications, the emotion generalization hypothesis and the emotion maintenance hypothesis further explore the impact of emotions on behavior, simply categorizing them into positive and negative types [16,17]. Individuals in a positive or pleasant emotional state tend to avoid taking risks when faced with decisions involving uncertainty, opting for more conservative and moderate choices to maintain their sense of well-being. In contrast, those in a negative emotional state are more inclined to engage in risk-taking behaviors as a means of escaping their unfavorable emotional condition. Therefore, this study selects negative emotions as the focus of investigation because individuals experiencing such emotions are more likely to exhibit risk-taking behaviors.

The Appraisal-Tendency Framework (ATF) theory proposes that different negative emotions have distinct impacts on behaviors [18]. Negative emotions can generally be categorized into six types: fear, anger, anxiety, disgust, embarrassment, and sadness [19]. The ATF theory posits that emotions with identical valence, such as fear and anger, may have contrary influences on decision-making and judgment [18]. Fear is a typical adaptive emotion that may lead individuals to respond behaviorally to potential stimuli [20]. Anger, on the other hand, is a psychological state that attributes unfavorable outcomes of the environment or situations to oneself or others. It often includes dissatisfaction or hostility in response to perceived harm, provocation, or threats [21]. The ATF theory elucidates that fear and anger emotions diverge in certainty and control when facing incidents [18]. Individuals exhibiting anger exhibit high certainty regarding events, attributing the incident predominantly to others as responsible. Conversely, those experiencing fear display low certainty, attributing the incident primarily to environmental factors [22]. This suggests that these two negative emotions may influence individual unsafe behaviors differently. Therefore, this study selects fear and anger as the independent variables to investigate their effects on unsafe behaviors. Current research on unsafe behaviors among construction workers primarily adopts an organizational perspective, focusing on factors such as safety climate [23], safety management [24] and safety performance [25]. However, studies addressing the individual emotional states of construction workers remain relatively scarce.

Physiological signals in response to external stimuli can recognize different emotional states. Generally, data used for emotion recognition can be categorized into two main types: (1) facial expressions and vocal tones, which are easy to collect but can be easily concealed or masked [10]; and (2) physiological indicators such as electroencephalogram (EEG), galvanic skin response (GSR), skin temperature (SKT), respiration (RSP), electrocardiogram (ECG), electromyogram (EMG), and other physiological signals, which are difficult to disguise. Emotion recognition based on physiological signals has gained significant attention due to its accuracy and resistance to manipulation [26]. Physiological data can accurately reflect emotional states, significantly enhancing the precision of emotion recognition. Among the physiological signals, EEG plays a dominant role in emotion-related research because emotions are closely linked to brain activity. Furthermore, the application of EEG-based emotion classification offers distinct advantages in the construction industry. Unlike traditional emotion recognition methods, such as self-reports or facial expression analysis, EEG enables real-time, objective, and precise monitoring of workers’ emotional states, which are crucial in high-risk environments [10,27]. By detecting negative emotional states that may impair judgment or lead to unsafe behaviors, EEG facilitates timely interventions to mitigate risks and enhance safety performance on construction sites [28]. The integration of EEG for monitoring emotional states thus represents a novel and effective strategy to improve worker safety and reduce the incidence of accidents. EEG’s objectivity makes it an effective tool for recognizing the emotions of construction workers.

The effects of emotions persist through residual mechanisms, in which emotions unrelated to the current task continue to influence decision-making in subsequent tasks, affecting individuals’ judgments and choices. Johnson and Tversky suggest that incidental emotions unrelated to decision-making can affect judgments about events with the same emotional valence, thereby further influencing decisions [17]. The concept of psychological capital was first introduced by the renowned American scholar Luthans in 2005 [29]. It refers to “a positive psychological state exhibited by individuals in life, surpassing the core elements of social capital and human capital, and serving as a psychological resource that promotes personal growth and enhances work performance”. Research on psychological capital has garnered widespread attention, and its impact on work performance has been validated [30].

**Figure 1:**
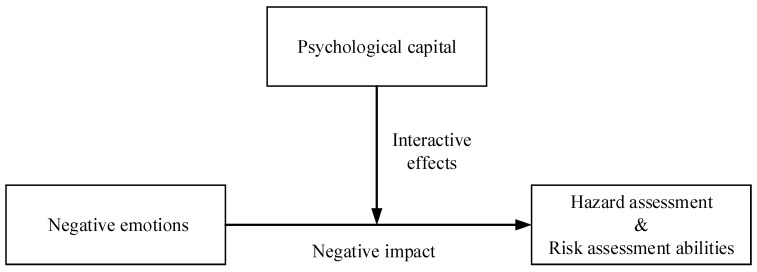
Conceptual framework of emotions, hazard assessment, and psychological capital.

The aim of this study is to investigate the impact of negative emotions on the unsafe behaviors of construction workers through an elaborate behavioral experiment. Real-time physiological data from EEG signals were collected. Three emotional states, namely fear, anger, and neutral emotion, were aroused, while safety hazard identification capability and risk assessment capability were employed as indicators of a worker’s ability to avoid unsafe behavior by effectively identifying hazards and assessing risks. The role of psychological capital in the process through which emotions impact hazard identification and risk assessment was thoroughly examined. The findings will contribute to improving onsite safety management through individual’s emotional stimulation and improvement. The relationships among emotions, hazard assessment, and psychological capital are illustrated in Figure 1. This conceptual figure outlines how emotions influence hazard assessment and risk assessment abilities, while psychological capital moderates the relationships between these variables.

## 2. Literature Review

### 2.1. The Impact of Emotions on Hazard Assessment

The effects of emotions involve a residual mechanism, where task-irrelevant emotions influence decision-making. The emotion generalization hypothesis, first proposed by Johnson and Tversky, suggests that incidental emotions unrelated to decision-making can affect individuals’ judgments of events with similar emotional valence, thereby influencing their decisions [17]. Specifically, positive emotions reduce individuals’ subjective risk assessments, making them more likely to engage in risk-seeking behaviors. Conversely, under negative emotions, individuals tend to make more pessimistic risk predictions, leading to a higher likelihood of risk-averse behaviors. The emotion maintenance hypothesis, proposed by Isen and Patrick, posits that individuals in positive or happy emotional states are generally unwilling to take risks when faced with risky decisions. Instead, they adopt more conservative and moderate choices to maintain their pleasant emotional state. In contrast, individuals in negative emotional states are more inclined to engage in risk-taking behaviors as a means to escape their negative emotional condition [16].

According to the Appraisal-Tendency Framework (ATF), incidental emotions generate judgments consistent with their appraisal components [18]. Under anger, individuals tend to evaluate situations through human agency attribution, characterized by high certainty and control, leading to less focus on environmental factors and an increased likelihood of unsafe behaviors. In contrast, under fear, individuals tend to evaluate situations through environmental attribution, resulting in greater attention to external factors and a reduced likelihood of unsafe behaviors.

These findings underscore the importance of understanding the effects of emotions on hazard assessment, particularly in high-risk industries such as construction, where emotional states can significantly impact safety outcomes.

### 2.2. The Interactions Between Psychological Capital, Emotions, and Hazard Identification and Risk Assessment

Psychological capital reflects an individual’s self-perception and self-esteem, representing a relatively stable psychological trait developed throughout life. It serves as a comprehensive indicator that reflects positive psychological states at work and predicts individual performance [31]. The four dimensions of psychological capital—optimism, hope, self-efficacy, and resilience—are characterized by their ability to promote positive individual behaviors, leading to higher performance outcomes.

The research findings indicate that psychological capital is positively correlated with job performance and negatively correlated with job stress [32]. Du and Yang, using Interpretive Structural Modeling (ISM), analyzed the relationships between psychological factors influencing safety performance. Their findings indicate that psychological capital and its various dimensions have a significant positive impact on the safety performance of civil aviation pilots [33]. Regarding the research on psychological capital in relation to unsafe behaviors, studies have found that psychological capital serves as an independent mediator and a chain mediator between miners’ unsafe behaviors and informal group cohesion. This indicates that enhancing individuals’ psychological capital can effectively reduce the frequency of unsafe behaviors among miners [34]. In the context of unsafe behaviors among construction workers, psychological capital has a positive impact on safety compliance, safety participation, and work engagement, suggesting that psychological capital may serve as a potential predictor of work engagement [35]. Furthermore, other studies have found that psychological capital positively influences both safety compliance and safety participation, indicating that organizations can reduce the occurrence of unsafe behaviors by enhancing individuals’ psychological capital [36].

This study uses safety hazard recognition ability and risk assessment ability to measure the likelihood of individuals effectively performing hazard identification and risk assessment. The stronger an individual’s safety hazard recognition and risk assessment abilities, the lower the likelihood of them engaging in unsafe behaviors. Conversely, weaker safety hazard recognition and risk assessment abilities increase the likelihood of engaging in unsafe behaviors.

### 2.3. EEG Applications to Construction Safety

Electroencephalography (EEG) is a technique for recording the electrical activity of the brain from the scalp. It utilizes precise electronic instruments to amplify and record spontaneous biological potentials from the brain, producing graphical representations of the spontaneous and rhythmic electrical activity of neural populations as captured by electrodes [37]. EEG signals comprise various brain wave rhythms associated with different frequency levels of brain activity, including delta waves (0.5–4 Hz), theta waves (4–7 Hz), alpha waves (8–13 Hz), beta waves (14–30 Hz), and gamma waves (>30 Hz).

In recent years, the application of EEG in safety behavior research has garnered increasing attention. Due to its ability to record signals in real-time, its non-invasive nature, wireless capabilities, and low cost, EEG can effectively analyze the unsafe psychological states of construction workers [38]. Ke et al. explored the safety behaviors of construction workers. They found that when workers were in states of anxiety and distraction, their response accuracy and reaction time significantly declined, increasing the risk of accidents. EEG monitoring discovered that β and γ activity in the left temporal lobe and right prefrontal cortex could distinguish between anxiety and distraction, particularly in channels T7 and AF4 [27]. Li et al. proposed an effective framework that comprehensively considers the impact of multiple critical unsafe psychological factors—namely acrophobia, distraction, and mental fatigue—on workers’ adverse mental states at heights. This framework aims to provide a holistic understanding of how these critical unsafe psychological factors influence workers’ mental conditions [38].

Additionally, EEG technology has been employed for emotion recognition and classification. A study has explored the combination of wavelet entropy and average wavelet coefficients as potential EEG features for classifying emotional valence and arousal [39]. Kolodyazhniy et al. applied pattern classification analysis to examine specificity. The analysis was conducted using Sequential Backward Selection (SBS) and Sequential Forward Selection (SFS) algorithms with different classifiers, including K-NN, neural networks, LDA, and QDA, along with four different cross-validation methods. The K-NN model, with 17 cross-validation methods, achieved the highest accuracy in both participant-dependent and stimulus-dependent classifications, reaching an accuracy of 77.5% for unknown objects under unknown conditions [40]. In the construction industry, recent studies have highlighted the potential of EEG technology for monitoring workers’ emotional states and their impact on safety behaviors. For instance, Athavipach et al. utilized ear-EEG to classify basic emotions using valence and arousal models, demonstrating the practicality of wearable EEG devices in real-world scenarios and their application to construction safety [41]. Similarly, Mir et al. discovered that varying types and levels of construction noise significantly impacted EEG signals, particularly in the frontal and temporal lobes, thereby altering workers’ emotional states [42]. These findings underscore the utility of EEG technology as a valuable tool for enhancing safety management in high-risk industries like construction.

These studies demonstrate that EEG provides robust data support for safety behavior research and offers new perspectives for practical safety management measures. Traditional safety management primarily relies on behavioral observation and training. In contrast, EEG monitoring allows for real-time assessment of workers’ psychological states, providing a basis for developing more personalized safety interventions. As EEG technology continues to advance, its application in real-time safety monitoring is expected to become more widespread, contributing to reduced accidents and improved workplace safety.

## 3. Materials and Methods

### 3.1. Participants

A total of 24 participants were recruited for this experiment on 8 October 2022, lasting for three days. In numerous studies examining the cognition of construction workers, participants from construction-related fields are often recruited for laboratory-based research [43,44]. Accordingly, this study selected participants with relevant backgrounds in majors such as Construction Engineering Management and Civil Engineering to ensure alignment with the cognitive processes and typical working conditions of professionals in the construction industry. The selected participants were undergraduate or graduate students majoring in construction engineering management, civil engineering, or related fields. Two participants were excluded due to experimental failures, leaving data from 22 participants, all of whom were male. This selection ensured the quality of EEG signals and reflected the predominance of males in the construction industry workforce. While the limited sample size may constrain the generalizability of the findings, it is consistent with the exploratory nature of this study and the technical challenges associated with EEG data collection, which often necessitates controlled conditions to ensure signal quality and minimize variability. For instance, a study examining high-altitude construction workers utilized a sample of 10 participants to assess the effects of interventions on emotional states and mental fatigue using EEG signals [45]. Similarly, another study investigating the impact of noise on cognitive performance in construction workers used 27 participants to examine the effects of noise exposure on attention, stress, and mental workload through EEG measurements [27]. Comparable sample sizes have been adopted in other EEG studies focused on emotion recognition, highlighting the practicality of such methodologies in laboratory-based research settings [46].

Table 1 lists the characteristics of the participants, including their age, average daily sleep duration, smoking status and frequency, alcohol consumption and frequency, number of times they received safety training, and number of safety knowledge lectures they attended. Overall, the participants had an average of approximately 8 h of sleep per day. Most participants reported low smoking frequency (mean = 1.18) and moderate alcohol consumption (mean = 1.68). On average, they attended safety training and safety knowledge lectures about 1.68 and 1.64 times, respectively.

### 3.2. Psychological Capital

The selection of psychological capital is based on the classical four-dimensional Psychological Capital Scale developed by Luthans et al. [31]. The scale has four dimensions: self-efficacy, hope, resilience, and optimism, with a total of 24 items, six for each dimension. Examples of items for each dimension are as follows. For self-efficacy, an example is “I am confident in my ability to analyze multiple solutions to complex problems”. For hope, an example is “I can discover various pathways to achieve my goals”. For resilience, an example is “When faced with adversity, I typically recover quickly”. Lastly, for optimism, an example is “I generally expect things to work out in my favor”. Each item is scored using a 5-point Likert scale, where a score of 1 indicates strong disagreement and a score of 5 indicates strong agreement. Higher scores reflect a greater level of psychological capital among participants. As shown in Table 2, the structure of the psychological capital questionnaire, including the number of questions, scoring method, and representative examples, provides a clear and concise overview of the assessment framework.

### 3.3. PANAS Self-Assessment Emotion Questionnaire

This experiment utilized the classic Positive and Negative Affect Schedule (PANAS) to measure the level of emotional arousal [47]. The PANAS employs a 5-point Likert scale to evaluate individuals’ emotional experiences, with higher scores indicating greater intensity of the corresponding emotional experience. Based on the original scale, this study simplified the questionnaire to align with the target emotions of the experiment, focusing on five basic emotional judgments: joy (pleasant, happy), interest (engaged, amused), fear (afraid), anger (angry), and calmness (peaceful, serene). Participants were required to rate their responses to descriptions of these specific emotions. As shown in Table 3, the structure of the simplified PANAS, including the number of questions, scoring method, and representative example, is summarized.

### 3.4. Safety Hazard Recognition and Risk Assessment

Safety hazard recognition and risk assessment are essential components of workplace safety, particularly in high-risk industries like construction. Safety hazard recognition involves identifying potential dangers in the environment, while risk assessment entails evaluating the likelihood and severity of those hazards to make informed decisions about mitigating risks. This study investigated the effects of emotions on safety hazard recognition and risk assessment by exposing participants to video clips designed to evoke specific emotional states. Two key indicators were used to assess safety hazard recognition: recognition accuracy (%), which measures the ability to correctly identify hazards in a series of images, and recognition response time (ms), which evaluates the time taken to make these assessments. Participants were asked to determine whether the presented images contained safety hazards, and their performance was analyzed under different emotional conditions to explore how emotions impact hazard recognition. For risk assessment, participants were presented with 36 hazard descriptions representing common safety hazards. They were instructed to rate the perceived risk associated with each description on a 5-point scale, where higher scores indicate a greater perceived risk. Additionally, risk assessment response time (ms) was recorded as an indicator of the cognitive effort and deliberation involved in evaluating the risks. This aspect of the study aimed to understand how emotional states influence the precision and thoroughness of risk assessment. By focusing on these two dimensions—safety hazard recognition and risk assessment—, this study provides insights into the cognitive processes underlying safety decision-making and highlights the impact of emotional states on these critical safety functions.

### 3.5. Experiment Procedures

The experiment consisted of two main parts: (1) the induction of target emotions and (2) tests for safety hazard identification and risk assessment ability. The experiment was conducted in a controlled environment where participants were seated comfortably in front of a computer. The experiment began with instruction and practice to familiarize participants with the procedure. The participants were asked to complete a Basic Personal Questionnaire (Q1) to capture demographic and personal background data, the Psychological Capital Questionnaire (Q2) to evaluate their psychological resilience, optimism, and other psychological capital traits, and the PANAS Self-Assessment Emotion Questionnaire (Q3) to evaluate their initial emotional state. EEG devices were then applied, and baseline EEG signals were recorded with the participants in two states—eyes open and eyes closed. This initial step ensured that any effects of baseline differences in these states were accounted for.

**Figure 2:**
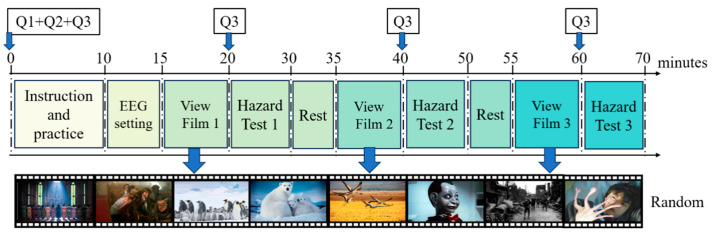
Experimental procedure diagram. Q1 is the Basic Personal Questionnaire, Q2 is the Psychological Capital Questionnaire, and Q3 is the PANAS Self-Assessment Emotion Questionnaire.

As shown in Figure 2, the participants were then exposed to a series of video clips designed to induce target emotional states. The clips included diverse themes and scenes to elicit various emotional responses. Following each film viewing, the participants were asked to complete an emotional state evaluation scale (using the PANAS Self-Assessment Emotion Questionnaire, labeled Q3) to assess their arousal and emotional states.

After the emotion induction phase, the participants proceeded to the safety hazard identification and risk assessment phase. This part of the experiment tested their ability to identify safety hazards and assess risks under the influence of the induced emotions. Each round consisted of 32 trials, divided into two types of tasks: 20 trials focused on safety hazard recognition, where the participants determined whether a safety hazard was present based on the presented stimuli, and 12 trials focused on risk assessment, where the participants rated the perceived risk of a specific unsafe behavior. A total of three rounds were conducted, with breaks offered between rounds to prevent fatigue or discomfort from the EEG device.

#### 3.5.1. Emotion Induction Material

Regarding studies on emotions and their influence on worker behavior, successful emotion induction is a prerequisite for conducting experiments. Common methods for inducing emotions include the following: (1) presenting the participants with emotionally charged images; (2) asking the participants to read sentences with strong emotional content; (3) showing the participants film clips or videos that evoke specific target emotions; (4) playing emotionally expressive non-verbal sounds or music to the participants [48]. National Institute of Mental Health (NIMH) scientists were among the first to develop a repository of emotion-inducing materials. This led to the development of the Affective Norms for English Texts (ANET) [49], the Affective Norms for English Words (ANEW) [50], the International Affective Picture System (IAPS) [51], and the International Affective Digital Sounds (IADS) [52].

Watching video clips involves both visual and auditory stimulation, combining the advantages of music and image induction [53]. The EEG devices were thoroughly calibrated before randomly playing pre-prepared neutral, fear, and anger emotion-inducing video clips while simultaneously recording the participants’ EEG signals. Each emotional state was selected based on the ratings provided by the participants, who evaluated the emotional impact of the chosen film clips [53]. The highest-scoring clips, referring to the clips that received the highest ratings for their effectiveness in evoking the target emotions, were used in the experiment and are listed in Table 4. To ensure consistency, the screen recording software was used to capture the selected film clips, and each clip was uniformly set to a length of 4–5 min. The resolution of the clips was set to 1280 × 760, and the video format was standardized to WMV. A total of 2–3 video clips were selected for each emotional state—neutral, fear, and anger—for the experiment.

#### 3.5.2. Safety Hazard Identification and Risk Assessment Test Material

In general, both prediction methods, which involve anticipating potential hazards based on past experiences, and review methods, which entail analyzing previous incidents to learn from them, can assist workers in enhancing their ability to identify risks. However, there is often a fundamental difference between imagined scenarios and real-world performance [54]. Therefore, this study selected photos from construction sites, using real-world settings to assess construction workers’ safety hazard identification and risk assessment abilities under different emotions. For safety hazard identification, this study adopts a behavioral experiment where participant were required to view construction site images and identify the presence of safety hazards [55]. This method effectively evaluates participants’ cognitive ability to recognize hazards. For risk assessment abilities, this study quantifies hazard perception by integrating perceived injury frequency and severity into a comprehensive evaluation score [56]. By integrating these established methodologies, this study enhances its ability to assess how emotional states influence both hazard identification and risk assessment in construction contexts. This approach provides a robust framework for understanding the impact of emotions on hazard recognition and risk assessment processes, which are critical determinants of safety performance in the construction industry. Safety hazard identification accuracy (RA, unit: %) and safety hazard identification response time (RT1, unit: milliseconds, hereinafter referred to as ms) were collected, reflecting the participants’ ability to identify safety hazards under the influence of emotions. These metrics help to explore whether emotions impact individual safety hazard recognition capabilities. The risk assessment score (RS, unit: points), calculated by summing the participants’ evaluations of identified hazards on a 1 to 5 scale (with 1 representing the lowest perceived risk and 5 representing the highest), and risk assessment response time (RT2, unit: ms), assessed how emotions may influence the participants’ risk assessment ability.

**Figure 3:**
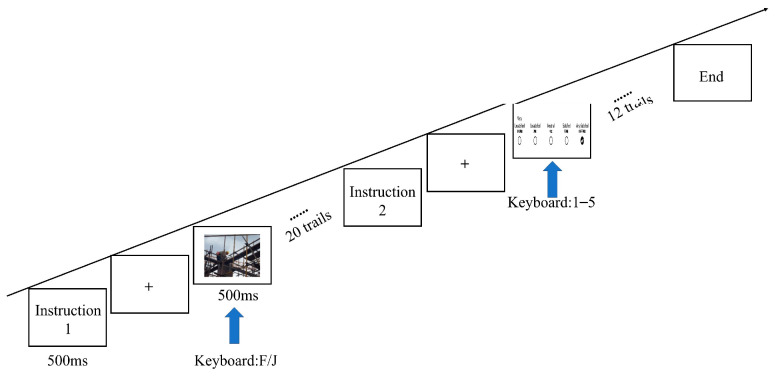
Safety hazard identification and risk assessment procedure.

The experiment was conducted using E-prime 2.0 software (version 2.0.10.356). The formal experiment consisted of 3 rounds (neutral, fear, and anger emotions), each containing 32 trials, including 20 trials for safety hazard identification judgment and 12 trials for risk assessment. In the safety hazard identification task, a total of 60 images were utilized from the image library, comprising 24 images without safety hazards and 36 images with safety hazards. For each trial, images were randomly selected from this pool, maintaining a presentation ratio of 2:3 between safe and hazardous images. The E-prime presentation for the safety hazard identification and risk assessment parts of the formal experiment is shown in Figure 3. At the start of the formal experiment, the instructions were presented, followed by a red fixation cross “+” (500 ms) while waiting for the participants’ judgment. The participants first made a safety hazard identification judgment based on whether there was a safety hazard in the image, responding with F (hazard present) or J (no hazard). Next, they rated the risk level of the hazardous scenario described in the text by pressing keys “1–5”, where each number corresponds to a specific level of perceived risk, ranging from minimal (1) to severe (5). Each number represents a corresponding score. This process prompted participants to make quick judgments. After making a judgment, a blank screen (300 ms) was shown, completing one trial, and then the next trial was presented after rest.

#### 3.5.3. EEG Data Collection

**Figure 4:**
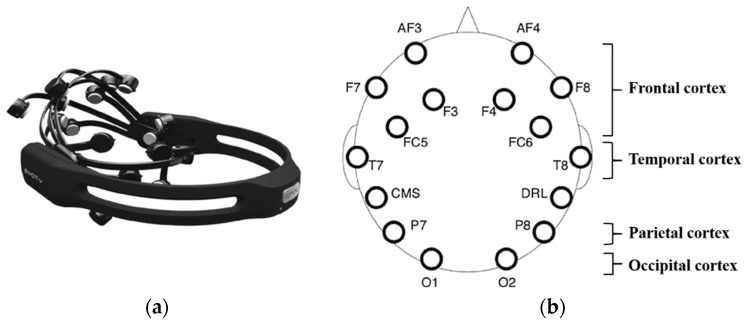
(**a**) EEG Collection Device; (**b**) EEG Channels.

The study employed the Emotiv EPOC X (Emotiv Inc., San Francisco, CA, USA) to capture real-time EEG signals from 22 participants across three emotional states (neutral, fear, anger), resulting in a total of 66 samples. The device recorded data at a sampling rate of 128 Hz, using 14 channels placed across different brain regions (Figure 4): the frontal lobe (AF3, AF4, F3, F4, F7, F8, FC5, FC6), temporal lobe (T7, T8), parietal lobe (P7, P8), and occipital lobe (O1, O2). During the data collection, the participants were asked to breathe slowly and evenly, keep their eyes closed, and minimize any physical movements to reduce interference.

### 3.6. Data Analysis Processing, Feature Extraction, and Analysis

#### 3.6.1. EEG Data Processing

To ensure accurate statistical analysis and address the sensitivity of EEG signals measured in microvolts (μV), we conducted preprocessing early in the emotion recognition phase. This was accomplished using the EEGLAB toolbox in MATLAB 2020, a widely recognized platform for EEG data analysis initially proposed by Delorme and Makeig in 2004 [57]. The toolbox facilitated essential preprocessing steps, including baseline correction, filtering, and independent component analysis (ICA), to minimize external interference and enhance data quality.

**Figure 5:**
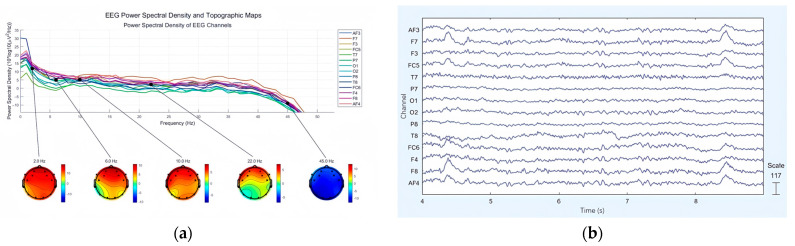
(**a**) Frequency domain power distribution of each channel; (**b**) EEG waveform after filtering.

First, we loaded the EEG data into EEGLAB, confirmed the electrode placements, and corrected for any baseline drift. We then applied a Hamming windowed sinc FIR filter to isolate the frequency range of 0.5 to 50 Hz, encompassing the main frequency bands associated with brain activity (delta (δ) (1–4 Hz), theta (θ) (4–8 Hz), alpha (α) (8–13 Hz), beta (β) (13–30 Hz), and gamma (γ) (31–47 Hz)), while reducing external noise. Additionally, a notch filter was used to eliminate interference from power lines. Finally, we performed ICA processing using the ADJUST toolbox (version 1.1) in EEGLAB to remove intrinsic artifacts, such as eye movements and facial muscle activity. Figure 5a presents the power spectral density of EEG signals, where each colored trace corresponds to the frequency spectrum of an individual channel. The accompanying scalp topography maps illustrate the distribution of power at specific frequencies, highlighting the efficacy of preprocessing steps in reducing noise. Figure 5b displays the time-domain EEG waveforms after preprocessing, with time (in seconds) plotted on the horizontal axis and channel names on the vertical axis. The waveforms exhibit smoother and artifact-free characteristics, demonstrating the successful removal of noise and the overall effectiveness of the preprocessing pipeline. These results confirm the improved quality of the cleaned EEG data, with 66 preprocessed samples prepared for subsequent analysis in emotion recognition research.

#### 3.6.2. EEG Feature Extraction and Machine Learning Approach

This study focused on the T7 and T8 channels from the occipital region to extract two key EEG characteristics: sample entropy (SampEn) and power spectral density (PSD). This selection was based on significant variations in EEG signal complexity and frequency domain across different emotional states [58]. Sample entropy quantifies the complexity and unpredictability of time-series EEG signals and has demonstrated superior performance in emotion recognition tasks [59]. It is calculated using the formula below:

(1) $$SampEn\left(m,r,N\right)=-\underset{N\to \infty }{\lim}log(\frac{A_{m}\left(r\right)}{B_{m}\left(r\right)}),$$

where m is the embedded dimension, r is the tolerance for accepting matches (often a percentage of the standard deviation), N is the length of the time series, A is the number of template matches of length m+1, B is the number of template matches of length m.

PSD is a measure used to describe the distribution of power (signal strength) of an EEG signal across different frequency components. To calculate the PSD for five frequency bands ((delta (1–4 Hz), theta (4–8 Hz), alpha (8–13 Hz), beta (13–30 Hz), and gamma (31–47 Hz)), we divided the collected data into 216 epochs, each lasting 1 s, using a fixed window technique. We then transformed the time-domain signals into frequency-domain signals with the Fast Fourier Transform (FFT). PSD was computed for specific frequency ranges using the following formula [60]:

(2) $$PSD(f)=\underset{T\to \infty }{lim}\;E\left[\frac{|\hat{X}(f){|}^{2}}{T}\right],$$

where T refers to the duration of the signal, $\hat{X}(f)$ is the Fourier transform of the signal, and f indicates the frequency range.

We created a two-dimensional feature matrix from the SampEn and PSD EEG characteristics. This matrix served as input indicators, improving emotion recognition accuracy by combining temporal complexity with energy distribution in the frequency domain.

We adopted a machine learning approach to classify emotional states based on the extracted EEG features. The K-Nearest Neighbor (KNN) algorithm was chosen for its simplicity, efficiency, and effectiveness in classification tasks, as well as its advantage of not requiring parameter estimation [61]. Using KNN, classification models were developed to differentiate between three emotional states: neutral, fear, and anger. The input features for the KNN algorithm included Sample Entropy (SampEn), which quantifies the complexity and unpredictability of EEG signals in the time domain, and Power Spectral Density (PSD), which represents the energy distribution of EEG signals across different frequency bands (delta, theta, alpha, beta, and gamma). These features were selected due to their ability to effectively capture both temporal and frequency-domain characteristics of EEG signals, which are essential for accurate emotion classification.

The KNN algorithm utilize these extracted features as inputs and assigns each sample to one of the three emotional categories—neutral, fear, or anger—based on the majority vote of its nearest neighbors in the feature space. The optimal value of k (number of neighbors) in this study was determined experimentally to ensure balanced classification performance across all emotional categories.

Combining SampEn and PSD as input features enhanced emotion recognition accuracy by leveraging both temporal complexity and frequency-domain energy distribution. As an interdisciplinary domain, machine learning integrates concepts from probability, statistics, and complex systems to improve computational efficiency in mimicking human learning processes. In this study, it played a pivotal role in structuring and partitioning EEG data for precise emotional state classification.

#### 3.6.3. Statistical Analysis

To comprehensively analyze the relationship between EEG features and emotional states, statistical analyses were performed using SPSS 23.0 (Statistical Product and Service Solutions, IBM, Armonk, NY, USA). Specifically, descriptive statistics were used to analyze basic trends and distributions to summarize the dataset. One-way ANOVA was used to compare group means across emotional states, identifying significant differences. Correlation analysis was conducted to explore relationships between EEG features and emotional states. Regression analysis examined predictive interdependencies among variables, highlighting the impact of EEG features on emotional classifications. A two-way ANOVA was conducted to analyze the interactive effects of psychological capital and emotional states, providing insights into how these two factors jointly influence the dependent variables. Furthermore, a multiple linear regression analysis was performed to evaluate the influence of personal factors on participants’ hazard identification and risk assessment.

This combination of preprocessing, feature extraction, machine learning, and statistical analysis provided a robust framework for understanding the emotional states of construction workers based on their EEG signals. These methodologies can potentially inform real-time monitoring systems and improve workplace safety and mental health interventions. The following section presents the experimental results, highlighting the influence of emotions on hazard identification and risk assessment abilities, as determined through EEG data analysis and behavioral experiments described above.

## 4. Results

### 4.1. Emotion Classification Based on EEG Signals

**Figure 6:**
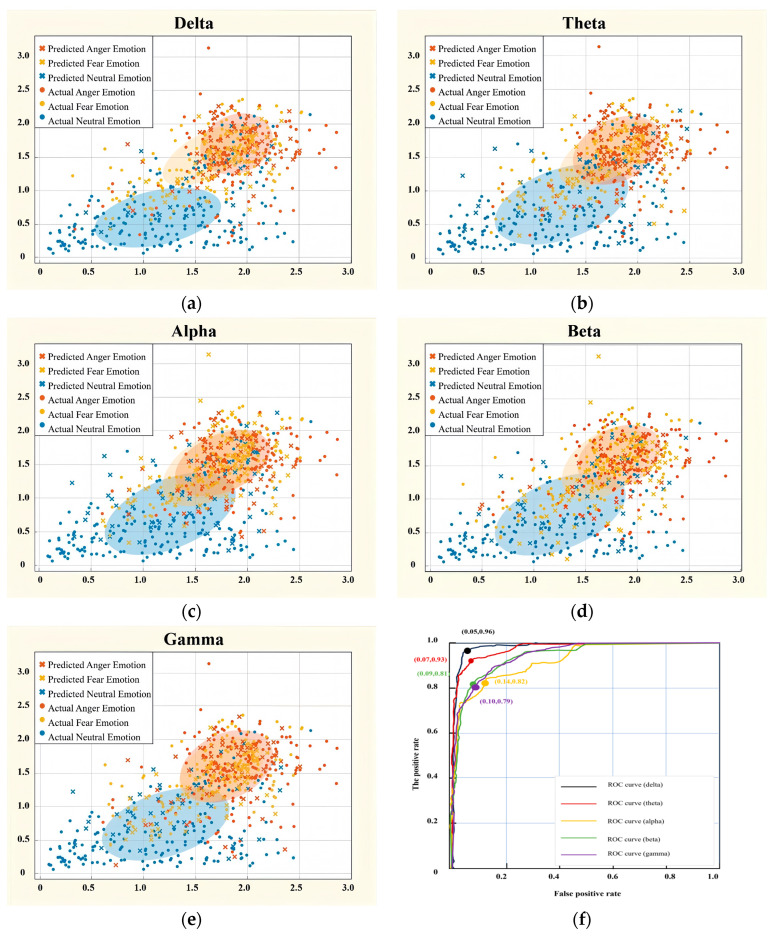
(**a**) Emotion classification scatter plot generated from features extracted from the delta (**a**), theta (**b**), alpha (**c**), beta (**d**), and gamma (**e**) frequency bands. (**f**) Receiver Operating Characteristic (ROC) curves for sentiment categorization by frequency band.

A dimensionality reduction algorithm was used to project the feature vectors into two dimensions using a randomly selected data subset to better assess the accuracy of the model’s classification. The classification results on each EEG band are shown in Figure 6. The scatter plot represents the classification results for the three emotional states (neutral, fear, and anger), each marked by a distinct color for clarity. Crosses (×) are used to indicate the predicted emotional states by the model, while circles (○) represent the actual emotional states based on the EEG data. The horizontal axis represents the first principal component, while the vertical axis represents the second principal component, both derived through dimensionality reduction. The position of the points reflects the projection of feature vectors in this reduced-dimensional space.

The comparison of different EEG frequency features reveals that delta exhibits better prediction accuracy across all classification algorithms (79%). Compared to features extracted from other frequency bands, delta has more predicted data points falling within the training set region, particularly evident in the effective classification of neutral emotions. The model correctly classified 208 samples as neutral (True Positive, TP = 96.3%), 162 samples as fear (TP = 75%), and 142 samples as anger (TP = 65.7%). In contrast, features from the alpha and gamma frequency bands demonstrate poorer classification performance, with the alpha band exhibiting weaker classification for anger emotions and the gamma band showing reduced classification accuracy for neutral emotions. The classification result for anger in the alpha band was poor, with only 122 samples correctly identified as anger (TP = 56.2%). This might be because the alpha band is usually linked to a relaxed state, which makes it less effective at capturing EEG patterns associated with anger. For the gamma band (Figure 6e), the scatter plot demonstrates significant overlap among the emotional states, resulting in lower classification accuracy. Specifically, the classification performance for neutral emotions was suboptimal, with only 171 samples correctly classified (TP = 78.8%). Similarly, the classification of anger emotions was less accurate, with 147 samples correctly identified as anger (TP = 67.1%). These findings underscore the gamma band’s limited capacity to effectively differentiate among the three emotional states based on the extracted EEG features. Given that the gamma band is associated with higher cognitive processes, such as information processing and memory functions, the observed overlap may stem from the fact that the three emotional states did not sufficiently engage these cognitive mechanisms. This could explain the difficulty in distinguishing EEG signals, particularly for neutral emotions, within the gamma band. Across all frequency bands, overlapping distributions of predicted and actual emotional states are observed in the reduced-dimensional space. A study on emotion classification using EEG signals highlighted that the overlapping neural features associated with different emotions pose significant challenges for accurate classification [62]. Similarly, another study emphasized that the overlapping characteristics of emotional states in EEG signals complicate the model’s ability to establish clear boundaries between categories [63]. These findings suggest that the inherent similarity of EEG features across certain emotional states, combined with the natural variability in brain activity, contributes to the classification challenges and limits the model’s capacity to delineate distinct emotional categories effectively.

To better analyze the classification performance of each EEG frequency feature, the Receiver Operating Characteristic (ROC) curves depicted in Figure 6f represent emotion classification performance using EEG signals across different frequency bands. The ROC curve for the delta band demonstrates the highest classification performance, with a point on the curve showing a true positive rate (TPR) of 0.96 and a false positive rate (FPR) of 0.05, indicating strong discrimination capability. The theta, alpha, beta, and gamma bands also perform well, with the theta band achieving a TPR of 0.93 at an FPR of 0.07. These results suggest that the delta band is particularly effective for classifying emotional states based on EEG signals, but all bands contribute valuable information for the classification task.

From the above classification results, the KNN algorithm can effectively classify the three emotions of the subjects (Table 5). The classification effect was best with 79.0% accuracy when we extracted the power spectrum and sample entropy feature of the delta band. In comparison, the classification effect was not good, with 69.3% accuracy when we used the power spectrum of the alpha band. Overall, the power spectra extracted from each frequency band are roughly between 70% and 80%, indicating that the model has achieved good classification results.

### 4.2. Influence of Emotional States on the Participants’ Hazard Identification and Risk Assessment Performance

**Figure 7:**
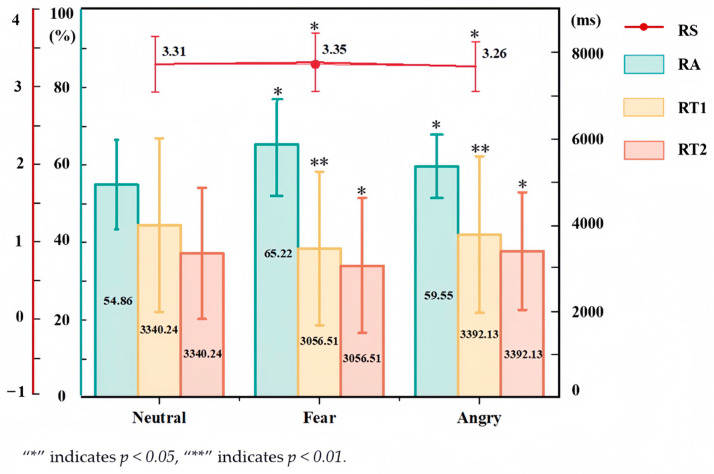
The results of unsafe behavior under different emotional states. RA indicates safety hazard identification accuracy (unit: %), RT1 indicates safety hazard identification response time (unit: ms), RS indicates risk assessment score (unit: points), and RT2 indicates risk assessment response time (unit: ms).

Each participant conducted hazard identification and risk assessment experiments under three emotions, and 132 sample data were collected. The relationships between emotions and the participants’ hazard identification and risk assessment performance were analyzed using the one-way ANOVA method. Figure 7 illustrates the statistical results of hazard identification and risk assessment performance under different emotional states.

Regarding safety hazard identification, there are significant differences in safety hazard identification accuracy (RA) and safety hazard identification response time (RT1) under different emotional states. This indicates that emotions affect safety hazard identification. The average RA under fear (65.22%) and anger (59.55%) is higher than those under neutral emotion (54.86%). This may be due to the heightened alertness of workers induced by fear and anger. Notably, RA is the highest fear. Furthermore, RT1 is shortest under fear (3456.11 ms), while it is longest under neutral (3996.92 ms). This suggests that fear may enhance workers’ alertness and attention, thereby improving their safety hazard identification capability.

Regarding risk assessment, the time and score of the risk assessment can better reflect workers’ risk perception ability. Analysis of the differences among the groups by the one-way ANOVA method, as presented (Figure 7), highlights significant differences in RS (*p* < 0.05). This suggests that emotions exert a substantial influence on the RS measures. The average RS is highest under fear (3.35) and lowest under anger (3.26), suggesting that risk perception ability is stronger under fear, whereas anger leads to a tendency to overlook risk signals. Additionally, the response times for risk assessment vary significantly under different emotional states. The shortest RT2 is observed under fear (3056.51 ms), and the longest RT2 under anger (3392.13 ms), indicating that risk can be perceived in a shorter time under fear, while more time is needed to perceive risks under anger. This might be related to the fact that anger induces negative emotions such as anxiety and stress, which interfere with the risk perception process.

### 4.3. Interactive Effects of Psychological Capital and Emotions

This study conducted a two-way ANOVA to analyze the interactions between psychological capital, emotions, and the participants’ hazard identification and risk assessment performance, verifying the role of psychological capital in moderating the impact of emotions on the participants’ hazard identification and risk assessment performance. Among the 22 subjects, the maximum score of psychological capital was 4.75, the minimum was 3.29, and the average score was 4.01. Psychological capital was categorized into two groups based on scores: a low psychological capital group (scores ranging from 3.29 to 4) and a high psychological capital group (scores ranging from 4 to 4.75), with 11 participants in each group. This categorization was used to facilitate the analysis of the influence of varying levels of psychological capital on the relationships between emotions and the participants’ hazard identification and risk assessment performance.

A randomized factorial analysis of variance was conducted with RT1, RA, RS, and RT2 as dependent variables, examining the participants’ hazard identification and risk assessment performance under different emotional states across the two groups with varying levels of psychological capital. The results are presented in Table 6. The main effect of the emotions is significant across RT1, RA, RS, and RT2 (*p* < 0.05).

For RA, emotions have a significant main effect (*p* = 0.026), particularly between the neutral and fear conditions. Psychological capital’s effect on RA is also significant (*p* = 0.046), with individuals exhibiting higher psychological capital showing slightly improved accuracy. The interaction between emotion and psychological capital is also significant (*p* = 0.034), suggesting that emotional states combined with varying levels of psychological capital influence RA. For RT1, there is a notable effect of emotions (*p* = 0.036), with anger causing slower response times than fear and neutral states. Psychological capital also significantly impacts RT1 (*p* = 0.025), with higher capital correlating with faster response times. The interaction of emotion and psychological capital has a significant effect here (*p* = 0.016), highlighting that individuals with high psychological capital mitigate the adverse effects of negative emotions like anger on reaction times. For RT2, emotions again have a significant main effect (*p* = 0.043), with fear eliciting the fastest response times in assessing risks. Psychological capital significantly affects RT2 (*p* = 0.038), where individuals with higher psychological capital respond more quickly. The interaction between emotion and psychological capital is also significant (*p* = 0.026), emphasizing that emotional states and psychological capital together influence reaction times during risk assessment. Notably, the interaction between emotion and psychological capital is significant for all indicators except RS. This suggests that the interaction between emotion and psychological capital does not significantly impact the RS indicator, potentially due to minimal variance among RS indicators.

The interaction between emotion and psychological capital is significant. Table 7 further analyzes the interaction effects of emotion and psychological capital on the RA, RT1, and RT2 indicators. Regarding RA, in the high psychological capital group, different emotional states have a significant impact on RA (F = 0.267, *p* = 0.034). In the low psychological capital group, RA also shows substantial differences across emotional states (F = 2.225, *p* = 0.001). For both high and low psychological capital groups, the effect of emotion on safety hazard identification capability is reflected in that RA in the fear group is consistently higher than RA in the anger group. Analyzing the impact of psychological capital on RA across different emotional groups, psychological capital shows a significant simple effect only in the fear group (F = 1.988, *p* = 0.028). In contrast, no significant simple effects are observed in the neutral (F = 0.003, *p* = 0.629) and anger groups (F = 0.674, *p* = 0.324). Although the moderating effect of psychological capital is not significant, when individuals are in a fearful emotional state, those with high psychological capital have a higher accuracy in hazard identification compared to those with low psychological capital. This suggests that psychological capital positively affects individuals’ safety hazard identification ability under fearful emotions. Individuals with high psychological capital, despite being in a negative emotional state, exhibit more stable psychological traits and are better able to identify safety hazards accurately.

In the high psychological capital group, different emotional states do not have a significant effect on safety hazard identification response time RT1 (F = 1.592, *p* = 0.28), indicating that individuals with higher psychological capital are better able to control their personal states, thus making RT1 less influenced by emotions. In the low psychological capital group, significant differences in safety hazard identification ability across different emotional states are observed (F = 1.613, *p* = 0.007), specifically with RT1 being greater in the anger group compared to the fear group. Analyzing the effect of psychological capital on RT1 across different emotional groups, psychological capital shows a significant simple effect only in the anger group (F = 2.562, *p* = 0.003). In contrast, no significant simple effects are observed in the neutral (F = 0.189, *p* = 0.685) and fear (F = 0.877, *p* = 0.762) groups. This suggests that in an angry emotional state, individuals with low psychological capital are more likely to be affected by anger, leading to decreased attention and impairing their ability to identify safety hazards, resulting in increased safety hazard identification response time.

Analysis of risk assessment response time reveals that the simple effect of emotion is significant within the low psychological capital group (F = 1.966, *p* = 0.009), but in the high psychological capital group, with no significant impact of different emotional states on RT2 (F = 1.713, *p* = 0.221). This reflects that individuals with higher psychological capital can better control their personal state, making RT2 less affected by emotions. Analysis of psychological capital’s effect on RT2 across different emotional groups shows significant simple impact of psychological capital in the fear group (F = 3.646, *p* = 0.004) and anger group (F = 1.412, *p* = 0.018). In contrast, the effect is not significant in the neutral group (F = 1.214, *p* = 0.452). In both anger and fear conditions, the risk assessment response time of the low psychological capital group is higher than that of the high psychological capital group, indicating that psychological capital has a negative effect on individuals’ risk assessment feedback time.

### 4.4. Influence of Personal Factors on the Participants’ Hazard Identification and Risk Assessment Performance

Standard multiple linear regression analysis was employed to explore the impact of personal factors on the participants’ hazard identification and risk assessment performance. The personal information collected includes smoking habits, alcohol consumption, sleep patterns, the number of safety training sessions attended, and the number of safety knowledge lectures attended. The results of the multiple linear regression analysis are presented in Table 8.

Based on the multiple linear regression analysis results, RA was significantly associated with the number of times receiving safety training (*p* = 0.002) and the number of safety knowledge lectures attended (*p* = 0.025). Furthermore, RT1 was significantly associated with average daily sleep duration (*p* = 0.045) and the number of times receiving safety training (*p* = 0.016). Specifically, RA had a positive correlation with the number of times receiving safety training and the number of safety lectures attended; meanwhile, RT1 had a negative correlation with both average daily sleep duration and the number of times receiving safety training.

Additionally, RS score was significantly associated with smoking status (*p* = 0.023), alcohol consumption (*p* = 0.004), and the number of safety knowledge lectures attended (*p* = 0.044). In contrast, RT2 was significantly associated with average daily sleep duration (*p* = 0.017) and the number of times receiving safety training (*p* = 0.024). RS had a negative correlation with smoking frequency and alcohol consumption and a positive correlation with the number of safety lectures attended. Similarly, RT2 negatively correlated with both average daily sleep duration and the number of times receiving safety training.

The findings indicate significant effects of emotions and psychological capital on safety behaviors. In the subsequent discussion, these results are examined within the context of existing theories, and their implications for safety management in the construction industry are explored.

## 5. Discussion

### 5.1. Negative Emotions Improve Risk Assessment Ability

This study confirms the emotion generalization hypothesis, which posits that individuals experiencing negative emotions are more pessimistic about risk prediction and, consequently, more likely to engage in risk avoidance behaviors. Driven by the motivation to avoid risk, these individuals enhance their ability to identify potential safety hazards and improve their risk assessment capabilities, thereby reducing the likelihood of engaging in unsafe behaviors. Our findings indicate that individuals experiencing fear align more closely with this hypothesis, as their ability to recognize potential safety hazards and assess risks was significantly higher than those in a neutral emotional state. Additionally, the multiple linear regression analysis results indicate that personal factors are significantly associated with individual safety hazard recognition capabilities and risk assessment capabilities.

Conversely, under the influence of anger, there was no significant difference in safety hazard recognition compared to a neutral emotional state, and risk assessment abilities were notably lower. This observation aligns more with the emotion maintenance hypothesis and the Affect Transfer Framework (ATF) evaluative tendency frame effect [64]. This finding is consistent with related studies by other scholars, suggesting that individuals in a negative emotional state are more inclined to engage in risky behaviors as a means to escape their current negative state [65,66]. This behavior increases certainty in subsequent decisions, thereby reducing the risk assessment of potential events to alleviate their current negative state.

### 5.2. The Dynamic Role of Negative Emotions in Workplace Safety

Regarding fear, many scholars have focused on its role in avoidance behavior. When associative learning occurs within circuits involving fear conditioning, the learning process itself is referred to as fear conditioning. According to Gray’s reinforcement sensitivity theory [67], negative emotions such as fear can easily activate the Behavior Inhibition System (BIS) and induce avoidance behaviors. In the workplace, fear is often closely associated with punitive measures, which typically result in negative emotions that serve to inhibit certain behaviors. Fear, as a bottom-up state, allows individuals to quickly mobilize prior experiences when facing new situations, thereby rapidly enhancing cognitive levels [68]. Therefore, in practical construction processes, appropriate punitive measures can induce a certain level of tension in construction workers, enabling them to better utilize fear reflexes when facing unexpected situations and thereby reducing unsafe behaviors.

Based on the Affect Transfer Framework (ATF) evaluative tendency frame effect theory, the influence of specific emotions on behavior is determined by six frame characteristics of emotions. Anger, as an emotion characterized by high certainty and high control, impacts risk assessment similarly to the positive emotion of joy, which also has high control and certainty [18]. According to the ATF model, under the influence of anger, individuals tend to attribute problems to personal factors, neglecting environmental evaluation and consequently reducing personal risk assessment capabilities. This leads to a higher likelihood of unsafe behaviors compared to neutral emotions. Therefore, considering the personal attribution and certainty characteristics of anger, it is essential to monitor construction workers for signs of anger, identify specific targets of their anger, and employ measures such as psychological counseling and relaxation rooms to help workers quickly dissipate anger and maintain a calm state, thereby reducing the likelihood of unsafe behaviors.

### 5.3. Psychological Capital Buffers the Negative Emotions

The findings of this study contribute to the existing literature on the influence of emotions and psychological capital on unsafe behavior. Consistent with previous research, the significant impact of emotions on hazard recognition (RA) and response times (RT1 and RT2) was evident, reinforcing the notion that emotional states can either enhance or impair cognitive and behavioral responses to safety threats [69]. At the same time, it has been validated that the conclusions drawn by previous researchers [25,70,71] regarding the significant correlation between personal factors and individual safety hazard recognition capabilities, as well as risk assessment capabilities, are accurate. However, this study extends prior work by highlighting the moderating role of psychological capital. Specifically, in high psychological capital groups, emotional states had less impact on RT1 and RT2, suggesting that individuals with higher psychological capital can better manage their responses, even under stress. This aligns with the findings of Avey et al. [72], who noted that psychological capital buffers against negative emotional impact.

## 6. Conclusions

This study targets construction workers, examining the influence of emotions on their hazard identification and risk assessment performance. Using E-prime psychology experiment design software, a behavioral experiment based on social cognitive neuroscience was developed to explore differences in safety hazard recognition and risk assessment abilities under anger, fear, and neutral emotional states. Additionally, through the processing of EEG data and the application of the KNN machine learning method, emotional classification was achieved.

The main findings of the research are as follows. (1) EEG signals provide the data foundation for emotion classification, with the KNN algorithm achieving a maximum classification accuracy of 79.0%. (2) The findings reveal that emotions significantly affect construction workers’ safety hazard identification and risk assessment abilities. (3) Additionally, a notable interaction effect exists between psychological capital and emotions on the accuracy of safety hazard identification, feedback time for safety hazard identification, and feedback time for risk assessment, underscoring the importance of considering both emotional states and psychological resources in occupational safety conte1xts. (4) Multiple linear regression analysis indicates that personal factors significantly impact people’s hazard identification and risk assessment performance. This paper focuses on negative emotions, expanding the scope of research on the impact of emotions on behavior. In the context of limited attention to group emotions among construction workers, the study broadens the understanding of how emotions influence behavior by applying emotional research to the field of safety management. This contributes to enhancing the existing theoretical framework of safety production management.

However, this study also has limitations, including a small sample size and limited emotional states. The small sample size may have limited the statistical power of the findings, potentially increasing variability and limiting the generalizability of the results to a broader population. This limitation emphasizes the need for cautious interpretation of the findings. It also highlights the importance of future research with larger sample sizes to enhance validation and improve the generalizability of the results. Specifically, the current study involved only male participants, which limits the generalizability of the findings. Future studies should incorporate female participants to investigate potential gender differences in emotional responses and safety behavior, thereby providing a more comprehensive understanding of these dynamics. Additionally, exploring other emotions, such as sadness and anxiety, and employing additional physiological measures (e.g., EOG, GRV, ECG) and advanced algorithms (e.g., AR, neural networks, random forests) could enhance the analysis and classification of emotional impacts on unsafe behavior. Furthermore, while this study focused on students in construction-related fields, future research should involve active professionals working under high-pressure conditions. These individuals may exhibit distinct emotional and behavioral patterns due to their real-world work experience and exposure to environmental stressors. Investigating such differences could yield deeper insights into the emotional influences on unsafe behaviors in practical construction settings.

## Figures and Tables

**Table 1:** Descriptive statistics of participants’ background information.

Characteristics	Group	N	Min	Max	Mean	SD
	Age	22	18	26	22.2	2.23
Personal factors	Average daily sleep duration	22	6	8	7.77	0.42
	Smoking status and frequency	22	1	4	1.18	0.65
	Alcohol consumption and frequency	22	1	3	1.68	0.56
	Number of times receiving safety training	22	1	3	1.68	0.56
	Number of safety knowledge lectures attended	22	1	3	1.64	0.65

**Table 2:** Overview of the psychological capital questionnaire.

The Questionnaire	Questions	Description	Example
Psychological Capital Questionnaire	24 questions	5-point Likert scale, where ‘5’ represents a ‘fully agree’, and ‘1’ illustrates a ‘completely disagree’. There are 3 reverse scoring questions.	I believe I can successfully complete my work. (1–5 points)

**Table 3:** Overview of the positive and negative affect schedule.

The Questionnaire	Questions	Description	Example
Positive and Negative Affect Schedule	5 questions	5-point Likert scale, a higher number indicates a stronger emotional experience.	Interested (1–5 points)

**Table 4:** Alternative emotion-inducing clips.

Target Emotion	Alternative Clips
Neutral	March of the Penguins, To the Arctic, Winged Migration
Fear	Dead Silence, Parasyte, House of Wax
Anger	The Flowers of War, City of Life and Death

**Table 5:** Emotion classification accuracy by EEG frequency band.

EEG Frequency Band	Delta Wave (1–4 Hz)	Theta Wave (4–8 Hz)	Alpha Wave (8–13 Hz)	Beta Wave (13–30 Hz)	Gamma Wave (31–47 Hz)
Accuracy	79.0%	75.9%	69.3%	77.8%	75.9%

**Table 6:** The ANOVA results of the participants’ hazard identification and risk assessment performance under different emotional states.

	Low Psychological Capital	High Psychological Capital	Emotions			Psychological Capital			Emotions × Psychological Capital		
RA	X ± SD	X ± SD	F	*p*	η^2^	F	*p*	η^2^	F	*p*	η^2^
Neutral	0.58 ± 0.15	0.60 ± 0.08	2.205	0.026 *	0.55	0.39	0.046 *	0.22	0.68	0.034 *	0.20
Fear	0.64 ± 0.18	0.66 ± 0.06									
Anger	0.58 ± 0.09	0.61 ± 0.07									
RT1	X ± SD	X ± SD	F	*p*	η^2^	F	*p*	η^2^	F	*p*	η^2^
Neutral	3821 ± 1571	4172 ± 2452	1.162	0.036 *	0.206	1.65	0.025 *	0.322	1.854	0.016 *	0.369
Fear	3336 ± 1395	3575 ± 2167									
Anger	4231 ± 2145	3314 ± 1360									
RS	X ± SD	X ± SD	F	*p*	η^2^	F	*p*	η^2^	F	*p*	η^2^
Neutral	3.42 ± 0.56	3.18 ± 0.64	1.083	0.047 *	0.432	2.634	0.002 *	0.659	1.088	0.536	0.124
Fear	3.52 ± 0.63	3.17 ± 0.59									
Anger	3.32 ± 0.62	3.18 ± 0.55									
RT2	X ± SD	X ± SD	F	*p*	η^2^	F	*p*	η^2^	F	*p*	η^2^
Neutral	3218 ± 1462	3461 ± 1643	1.315	0.043 *	0.387	1.172	0.038 *	0.452	1.415	0.026 *	0.341
Fear	2797 ± 1714	3315 ± 1436									
Anger	3541 ± 1391	3243 ± 1385									

RA indicates safety hazard identification accuracy, RT1 indicates safety hazard identification response time, RS indicates risk assessment score, and RT2 indicates risk assessment response time; “*” indicates *p* < 0.05.

**Table 7:** Analysis of the main effects of emotion and psychological capital on RA, RT1, and RT2.

Source of Variation			df	MS	F	*p*	Post Hoc Comparison
RA	Emotions	High psychological capital	2	0.028	0.267	0.034 *	Fear group > Anger group
		Low psychological capital	2	0.689	2.225	0.001 *	Fear group > Anger group
	Psychological Capital	Neutral	1	0.122	0.003	0.629	
		Fear	1	0.242	1.988	0.028 *	High psychological capital > low psychological capital
		Anger	1	0.139	0.674	0.324	
RT1	Emotions	High psychological capital	2	2,206,447	1.592	0.28	
		Low psychological capital	2	3,597,751	1.613	0.007 *	Anger group > Fear group
	Psychological Capital	Neutral	1	679,098	0.189	0.685	
		Fear	1	312,745	0.877	0.762	
		Anger	1	4,629,750	2.562	0.003 *	Low psychological capital > high psychological capital
RT2	Emotions	High psychological capital	2	136,140	1.713	0.221	
		Low psychological capital	2	1,529,139	1.966	0.009 *	Anger group > Fear group
	Psychological Capital	Neutral	1	324,057	1.214	0.452	
		Fear	1	1,475,575	3.646	0.004 *	Low psychological capital > high psychological capital
		Anger	1	487,693	1.412	0.018 *	Low psychological capital > high psychological capital

RA indicates safety hazard identification accuracy, RT1 indicates safety hazard identification response time, RS indicates risk assessment score, and RT2 indicates risk assessment response time; “*” indicates *p* < 0.05.

**Table 8:** The multiple linear regression coefficients of personal factors on RA, RT1, RS, and RT2.

Type	Personal Factors	B	t	Sig.	VIF
RA	Constant	0.646	2.842	0.012	
	Average daily sleep duration	0.011	0.296	0.771	1.66
	Smoking status and frequency	0.002	0.037	0.971	1.409
	Alcohol consumption and frequency	−0.015	−0.639	0.003 **	1.241
	Number of times receiving safety training	0.047	0.93	0.002 **	2.098
	Number of safety knowledge lectures attended	0.001	0.022	0.025 *	1.995
RT1	Constant	4758.837	1.392	0.184	
	Average daily sleep duration	−723.531	−1.256	0.228	1.66
	Smoking status and frequency	−163.433	0.199	0.045 *	1.409
	Alcohol consumption and frequency	292.155	0.817	0.427	1.241
	Number of times receiving safety training	−1102.32	−1.455	0.016 *	2.098
	Number of safety knowledge lectures attended	425.349	0.667	0.065	1.995
RS	Constant	2.643	1.745	0.001	
	Average daily sleep duration	−0.09	−0.352	0.023 *	1.66
	Smoking status and frequency	0.065	0.179	0.861	1.409
	Alcohol consumption and frequency	0.178	1.126	0.278	1.241
	Number of times receiving safety training	−0.103	−0.306	0.764	2.098
	Number of safety knowledge lectures attended	0.222	0.785	0.044 *	1.995
RT2	Constant	284.09	0.093	0.927	
	Average daily sleep duration	−265.006	−0.517	0.613	1.66
	Smoking status and frequency	−1036.06	1.415	0.017 *	1.409
	Alcohol consumption and frequency	441.982	1.389	0.185	1.241
	Number of times receiving safety training	−765.426	−1.134	0.024 *	2.098
	Number of safety knowledge lectures attended	350.89	0.618	0.546	1.995

“*” indicates *p* < 0.05, “**” indicates *p* < 0.01.

## Data Availability

The datasets used in this study are not available as the ethical consent provided by participants does not permit use of the data beyond the current study. The publication of these data does not compromise the anonymity of the participants or breach local data protection laws.
